# Early critical cortical infarction by anti-angiotensin II type 1 receptor antibody: A case report and literature review

**DOI:** 10.1097/MD.0000000000025958

**Published:** 2021-05-28

**Authors:** Jeong-Hoon Lim, Man-Hoon Han, Yong-Jin Kim, Seung Huh, Chan-Duck Kim

**Affiliations:** a Department of Internal Medicine; b Department of Pathology; c Department of Surgery, School of Medicine, Kyungpook National University, Kyungpook National University Hospital, Daegu, South Korea.

**Keywords:** angiotensin II type 1 receptor antibody, antibody-mediated rejection, cortical infarction, kidney transplantation

## Abstract

**Rationale:**

Anti-angiotensin II type 1 receptor antibodies (AT_1_R-Abs) have been demonstrated to increase the risk of antibody-mediated rejection. We report a case of AT_1_R-Ab mediated rejection which caused early critical cortical infarction.

**Patient concerns:**

A 52-year-old man with end-stage kidney disease underwent preemptive kidney transplantation (KT) from his wife. He had no immunologic risk except ABO incompatibility. Proper desensitization treatment were applied prior to KT. On postoperative day 1, he showed stable clinical course with adequate urine output, but there was no decrease in serum creatinine level and imaging studies showed hypoperfusion in the transplanted kidney.

**Diagnoses:**

Allograft biopsy revealed total cortical infarction with severe necrotizing vasculitis, but the medullary area was preserved. Serum AT_1_R-Ab concentration was elevated from 10.9 U/mL before KT to 19.1 U/mL on 7 days after KT.

**Interventions:**

He was treated with plasmapheresis, intravenous immunoglobulin, rituximab, high-dose methylprednisolone, and bortezomib.

**Outcomes:**

The treatment showed a partial response, and he was discharged with 7.3 mg/dL creatinine level. At 4 months, his creatinine plateaued at 5.5 mg/dL and AT_1_R-Ab decreased to 3.6 U/mL.

**Lessons:**

This case highlights the risk of early active antibody-mediated rejection by preformed AT_1_R-Ab, suggesting its ability to exhibit atypical histopathologic findings, such as total cortical infarction.

## 1 Introduction

Antibody-mediated rejection (ABMR) remains a major contributor for graft loss in kidney transplant recipients (KTRs), and the diagnosis and treatment of ABMR remains a challenge.^[1]^ The positivity for direct crossmatches and the presence of donor-specific human leukocyte antigen (HLA) antibodies are well-known ABMR predictors, but the importance of non-HLA antibodies has also recently been increasingly reported.^[2]^ In this report, we present a case of a KTR who suffered aggressive ABMR in the early period of transplant caused by a non-HLA antibody against angiotensin II type 1 receptor (AT_1_R).

## 2 Case presentation

A 52-year-old Korean male with end-stage kidney disease caused by chronic glomerulonephritis underwent a preemptive living donor kidney transplantation (KT) from his wife in 25th July 2019. He had comorbid hypertension and diabetes, with a daily urine volume before KT being > 1 liter per day. Panel-reactive antibody levels were 2% and 0% for class I and class II, respectively (highest mean fluorescence intensity level: 303); no pretransplant donor-specific anti-HLA antibodies were noted. Pretransplant crossmatches were also negative for both T and B cells. The recipient was blood group A, while the donor was blood group B, and the recipient's baseline anti-B immunoglobulin G (IgG) antibody titer was 32, measured by the column agglutination technique. To remove the anti-B antibody, the patient underwent desensitization therapy (rituximab, plasmapheresis, and low-dose intravenous immunoglobulin [IVIG]) according to our center's desensitization protocol.^[3]^ The patient also started on standard triple immunosuppression 10 days before KT. After three sessions of plasmapheresis, the anti-B IgG titer decreased to 8, and the patient received 20 mg basiliximab induction because his immunologic risk was not high.

**Figure 1:**
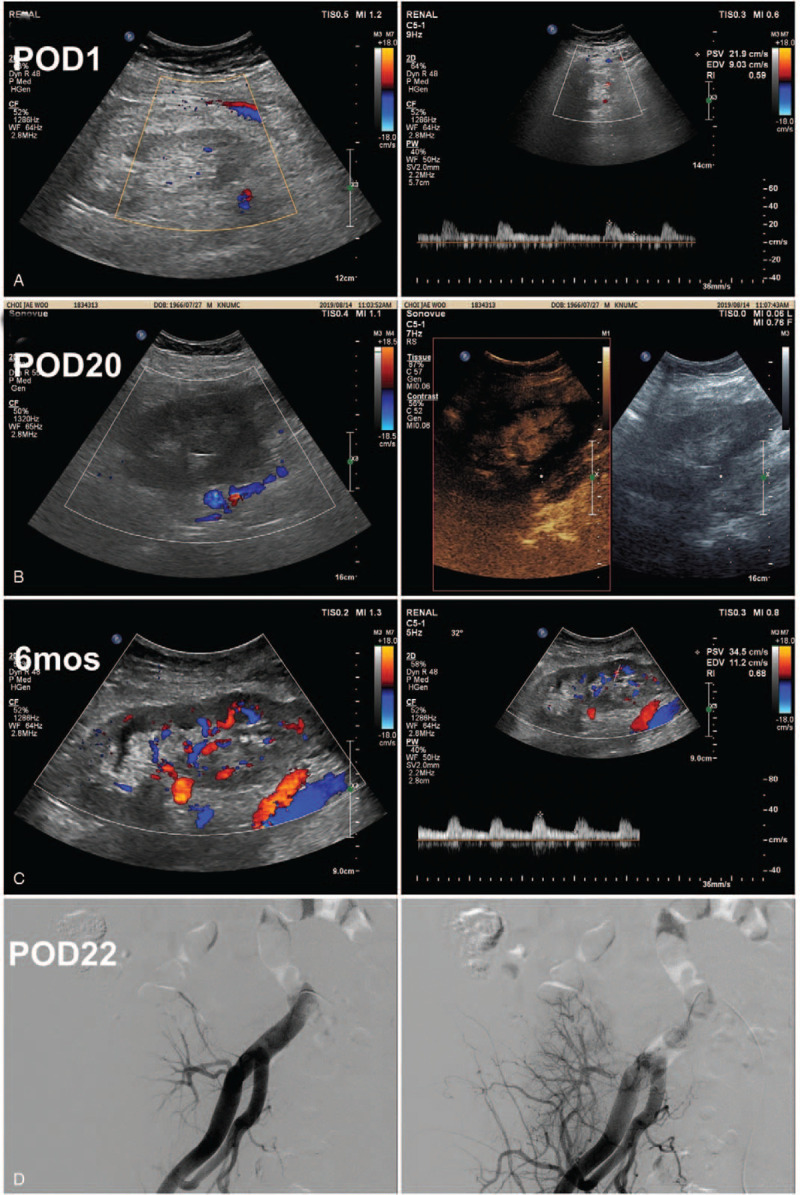
Changes of graft perfusion in graft Doppler sonography and graft angiography. (A) Decreased intrarenal perfusion with normal resistive index on postoperative day 1. (B) Decreased intrarenal perfusion, particularly in the cortical area, by allograft contrast-enhanced ultrasound on postoperative day 20. (C) Improved intrarenal perfusion with normal resistive index on the sixth postoperative month. (D) Right external iliac angiographic images on postoperative day 22. Graft renal arterial flow was good, but no cortical perfusion was noted.

**Figure 2:**
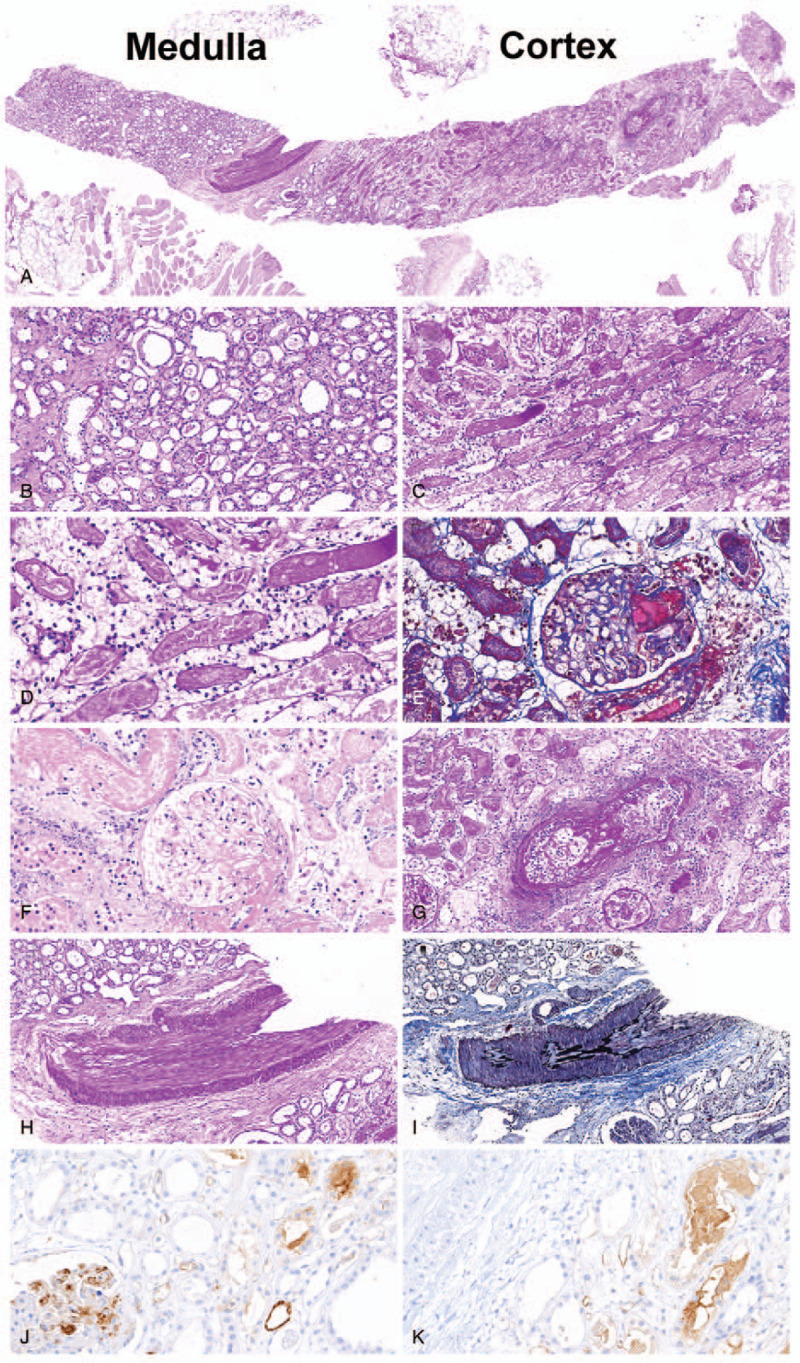
Histopathologic findings on postoperative day 6. (A) Cortical infarction with coagulative-type necrosis and preserved medulla (PAS; original magnification × 20). (B) Preserved medulla (PAS; original magnification × 200). (C, D) Cortical infarction with coagulative-type necrosis (PAS; original magnification × 200 and × 400, respectively). (E) Thrombi in glomerulus (electric trichrome; original magnification × 400). (F) Infarcted glomerulus (H&E; original magnification × 400). (G) Severe necrotizing vasculitis (PAS; original magnification × 200). (H, I) Arterial sclerosis (PAS and electric trichrome; original magnification × 100). (J, K) C4d staining by immunohistochemistry (original magnification × 400). H&E = hematoxylin and eosin, PAS = periodic acid-Schiff.

The cold ischemic time was 66 minutes because the donor had double renal arteries, thus needing common channel formation. During the operation, the patient blood pressure was maintained above 120/70 mmHg, and immediate graft function was good with an hourly urine output of > 200 mL. The total urine output was 5,400 mL during the first 24 hours following KT, and anti-B IgG antibody titer decreased to 2 and tacrolimus trough level 3.5 mg/dL on postoperative day (POD) 1. Although the patient showed a stable clinical course, his serum creatinine level did not decrease within the first 24 hours (remained 6.5 mg/dL), and his DTPA scan showed almost nonvisible tracer uptake in the transplant kidney. Graft Doppler sonography also revealed hypoperfusion with normal resistive index in transplant kidney, but the graft renal artery was well-perfused (Fig. 1A). So, tacrolimus was immediately stopped, and antithymocyte globulin (1.5 g/kg for 4 days) was started, considering acute tubular necrosis or acute tacrolimus nephrotoxicity. His urine volume was maintained above 3,000 mL/d; however, serum creatinine level was gradually increased to 8.3 mg/dL, and the platelet count was decreased to 57 × 10^9^/L on POD 5. Anti-B IgG titer was maintained to 2, with the absence of donor-specific anti-HLA antibodies. Graft biopsy was performed on POD 7, and the specimen showed a totally infarcted cortex with a preserved medulla (Fig. 2). The entire cortical tubulointerstitium showed a coagulative-type necrosis, and infarction of all glomeruli was also noted, so microvascular inflammation, such as peritubular capillaritis and glomerulitis, was difficult to assess. Diffuse and severe arteritis with necrosis (v3) was also seen, as well as thrombi in small arteries; donor-related arteriosclerosis (as3) was also observed. Although glomerular inflammation was difficult to assess because of infarction, some glomeruli had thrombotic microangiopathy. Peritubular capillaries were C4d+ by immunohistochemistry (C4d3).

**Figure 3:**
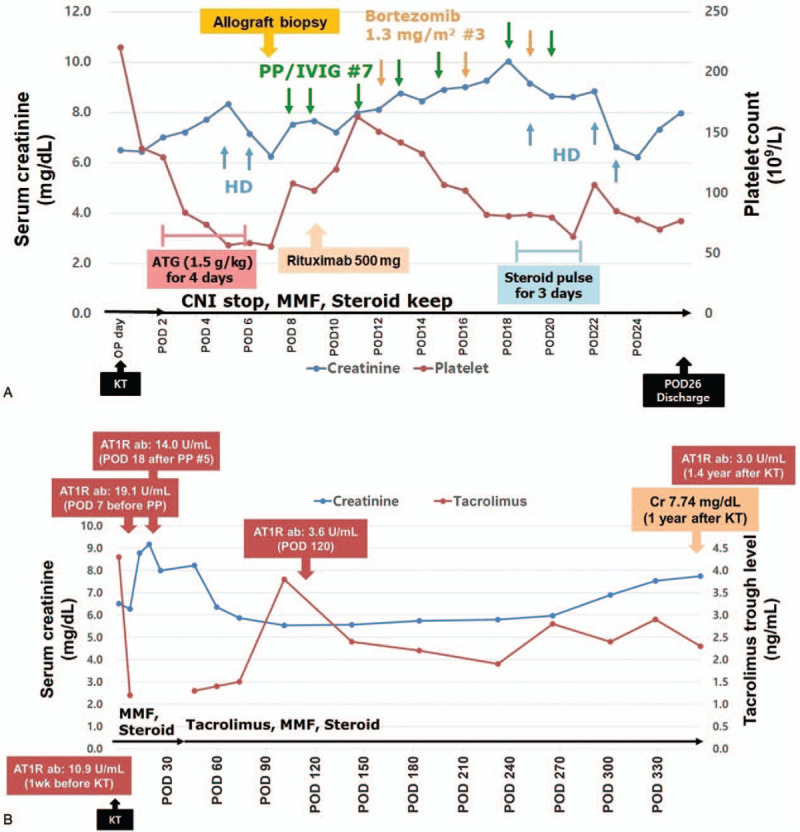
Clinical course and treatment. (A) In-hospital clinical course following kidney transplantation. (B) 1-year clinical course following kidney transplantation. AT1R = angiotensin II type 1 receptor, CNI = calcineurin inhibitor, IVIG = intravenous immunoglobulin, KT = kidney transplantation, MMF = mycophenolate mofetil, OP = operation, POD = postoperative day, PP = plasmapheresis.

Through these results, we considered thrombotic microangiopathy or active ABMR caused by non-HLA antibodies. Next, we performed CFH gene analysis, and no mutation was seen. With increased possibilities of active ABMR, we treated the patient using plasmapheresis (total 7 sessions every other day) with low-dose IVIG (0.1 g/kg after each plasmapheresis), rituximab (500 mg once), bortezomib (1.3 mg/m^2^ for 3 times), and high-dose methylprednisolone (500 mg for 3 days) (Fig. 3A). From the fifth day of ABMR treatment, his renal function gradually improved, but follow-up DTPA scan and Doppler sonography showed persistent hypoperfusion in the entire transplant cortical area (Fig. 1B). Graft angiography on POD 22 revealed good transplant renal arterial flow but little cortical perfusion (Fig. 1D).

**Figure 4:**
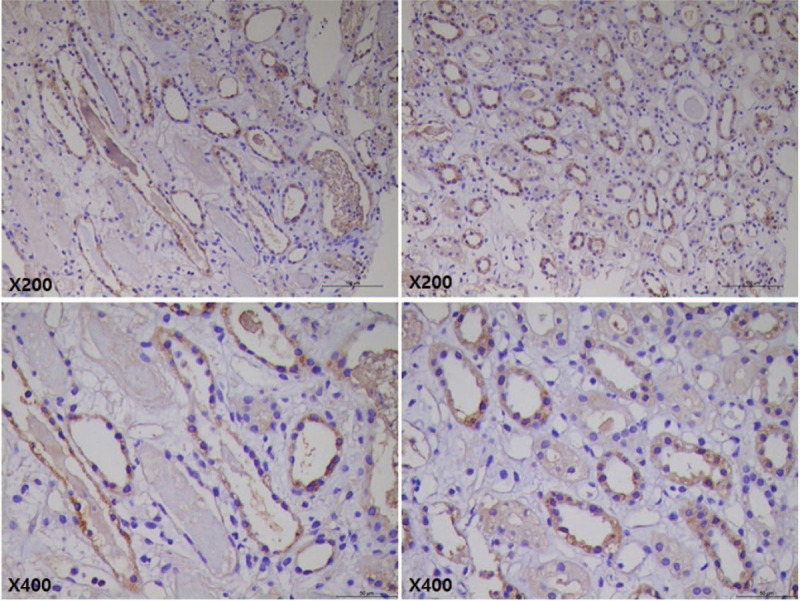
Immunohistochemical staining for angiotensin II type 1 receptor. Increased expression of angiotensin II type 1 receptor in overall tubules. Original magnification, × 200 and × 400 in the upper and lower rows, respectively.

In the course of treatment, we tried to find preformed non-HLA antibodies. Anti-major histocompatibility complex class I-related chain A antibody and anti-endothelial cell antibody were examined, and the results were negative. On the other hand, anti-AT_1_R antibody concentration was elevated from 10.9 U/mL before KT to 19.1 U/mL on POD 7. After five sessions of plasmapheresis for ABMR treatment, anti-AT_1_R antibody concentration was decreased to 14.0 U/mL. Graft biopsy specimen was also retrospectively stained for AT_1_R, and the specimen revealed an increased expression of AT_1_R on tubular cells (Fig. 4).

He was discharged on POD 26 with serum creatinine level of 7.3 mg/dL. At 4 months, his serum creatinine level plateaued at 5.5 mg/dL, and his serum anti-AT_1_R antibody level decreased to 3.6 U/mL. At 6 months, graft Doppler sonography showed an increased intrarenal perfusion with a normal range of resistive indices (Fig. 1C). The patient's graft function has decreased slowly, with a serum creatinine level of 7.7 mg/dL on the first postoperative year (Fig. 3B).

## 3 Discussion

We present an early aggressive ABMR case triggered by a preformed anti-AT_1_R antibody, and the patient had unusual histopathologic findings, such as cortical infarction and severe vasculitis. Despite immediate treatment for ABMR, the patient's graft function had not been fully recovered and nearly progressed to graft failure on the first postoperative year. Our case suggests the preformed anti-AT_1_R antibody increased the risk of ABMR and graft failure in the early period of KT even in patients without high immunologic risk.

Anti-AT_1_R antibodies are reported to be associated with arteritis or glomerulitis on allograft biopsy and elevated pro-inflammatory cytokine levels, which may cause vascular inflammation and further vascular rejection.^[4]^ Dragun et al. firstly reported the refractory rejection without donor-specific anti-HLA antibody, and the patients characteristically had endarteritis and vascular necrosis.^[5]^ Since then, subsequent studies have confirmed anti-AT_1_R antibodies’ correlation with an increased risk of ABMR and poor graft survival.^[6,7]^Table 1 summarizes the results of AT_1_R antibody studies in KT. Reinmoen et al. reported close association between C4d negative ABMR and AT_1_R antibody,^[6]^ and Giral and Lee et al. showed the positive association of pre-KT AT_1_R antibody with acute rejection.^[7,8]^ However, Deltombe et al. and Pinelli et al. reported that there was no association between AT_1_R antibody and graft outcome.^[9,10]^ Although the clinical relevance of AT1R antibody is still not clear, our case emphasizes that it can cause early fatal graft outcome.

**Table 1:** Summary of AT_1_R antibody studies in kidney transplantation.

Study	Number of recipients	Time of AT_1_R-Ab measurement	Cutoff value (U/mL)	Summary
Reinmoen et al. 2010^[6]^	97	Pre- and post-KT	17	85.7% of ABMR patients without C4d had AT_1_R-Ab.
Giral et al. 2013^[7]^	599	Pre-KT	10	Pre-KT AT_1_R-Ab was a/w early acute rejection.
Lee et al. 2017^[8]^	166	Pre-KT	9.05	Pre-KT AT_1_R-Ab was a/w acute rejection.
Philogene et al. 2017^[2]^	70	Post-KT	10, 17	AT_1_R-Ab levels were higher in ABMR patients.
Deltombe et al. 2017^[9]^	940	Pre-KT	10, 17	No association between pre-KT AT_1_R-Ab and graft outcome.
Pinelli et al. 2017^[10]^	142	Pre- and post-KT	17	No association between pre-KT AT_1_R-Ab and graft outcome.

In the immunohistochemical staining for AT_1_R, AT_1_R expression was increased in tubular cells, but the microvascular expression was not significant, which was similar with the previous study that reported the early ABMR by non-HLA antibody against vimentin.^[11]^ Tubular vimentin expression was increased at the time of ABMR, but peritubular capillary and podocyte vimentin expression was not changed before and after ABMR. Although the AT_1_R microvascular expression was not increased, an upregulated tubular AT_1_R expression would be enough to induce antibody-mediated vascular rejection via preformed anti-AT_1_R antibodies.

Cortical infarction in transplant kidney, especially involving the entire cortex, is not a common condition. Most cases of graft infarction result from the thrombotic or embolic occlusion of graft renal artery, prolonged transplant ischemia, perioperative bleeding, coagulopathies, and anatomic complexity.^[12]^ Our patient maintained a normal range of blood pressures during the operation and had high blood pressure during the postoperative period; the amount of perioperative bleeding was small. The urine output was also adequate immediately after declamping graft renal artery. At first, we considered the possibility of calcineurin inhibitor toxicity. However, stopping the calcineurin inhibitor medication was not effective in this patient. The histopathologic findings were not typical for ABMR and the patient had no donor-specific anti-HLA antibody, so ABMR was difficult to diagnose. After multidisciplinary meetings, we initiated ABMR treatment before non-HLA antibody confirmation, so as not to miss the golden time. Even with ambiguous microvascular inflammation, severe necrotizing vasculitis and acute thrombotic microangiopathy suggested ABMR. Lefaucheur et al. have proposed a new classification of antibody-mediated vascular rejection via a population-based study.^[13]^ They emphasize the importance of arteritis and KTRs with ABMR with vasculitis showed worse graft survival. However, there are few studies on vascular rejection treatment and targeted therapy for non-HLA anti-AT_1_R antibodies. Plasmapheresis or immunoadsorption is used to reduce anti-AT_1_R antibody titers^[14]^; rituximab and IVIG may have effects by lowering antibodies and reducing intrarenal complement activation.^[15]^ We applied these treatments, but their effect was not good, so further studies for vascular rejection treatment are required to improve the outcome.

In this case report, we highlight the risk of early critical ABMR by anti-AT_1_R antibody. Histopathologic findings showed total cortical infarction with coagulative-type necrosis and severe necrotizing vasculitis, but the medullary area was preserved. It was difficult to diagnose ABMR during biopsy, because no donor-specific anti-HLA antibody was noted and the histopathology was atypical for ABMR. If the patient shows cortical infarction with severe vasculitis and no specific immunologic and anatomical risks, the possibility of ABMR by non-HLA anti-AT_1_R antibody should be considered, and if suspected, aggressive treatment should be performed as soon as possible.

## Author contributions

**Conceptualization:** Jeong-Hoon Lim, Chan-Duck Kim.

**Data curation:** Jeong-Hoon Lim, Man-Hoon Han, Yong-Jin Kim, Seung Huh.

**Investigation:** Jeong-Hoon Lim, Chan-Duck Kim.

**Methodology:** Jeong-Hoon Lim.

**Visualization:** Jeong-Hoon Lim, Man-Hoon Han, Yong-Jin Kim.

**Writing – original draft:** Jeong-Hoon Lim, Chan-Duck Kim.

**Writing – review and editing:** Jeong-Hoon Lim, Chan-Duck Kim.
